# Risk perception among Brazilian individuals with high risk for colorectal cancer and colonoscopy

**DOI:** 10.1186/1897-4287-9-4

**Published:** 2011-07-28

**Authors:** Erika M Santos, Maria TC Lourenço, Benedito M Rossi

**Affiliations:** 1 Graduation Program, Antonio Prudente Foundation, Sao Paulo, Brazil

## Abstract

**Background:**

Risk perception is considered a motivating factor for adopting preventive behaviors. This study aimed to verify the demographic characteristics and cancer family history that are predictors of risk perception and to verify if risk perception is a predictor of colonoscopy adherence.

**Methods:**

Individuals with a family colorectal cancer history as indicated by a proband with cancer were interviewed by telephone. They responded to a questionnaire covering demographic characteristics, colonoscopy history and four questions on risk perception. Tests of multiple linear regression and logistic regression were used to identify associations between dependent and independent variables.

**Results:**

The 117 participants belonged to 62 families and had a mean age of 45.2 years. The majority of these individuals were female (74.4%) and from families who met the Amsterdam Criteria (54.7%). The average risk perception was 47.6%, with a median of 50%. The average population perception of individual risk was 55.4%, with a median of 50%. Variables associated with a higher risk perception were age, gender, religion, school level, income, and death of a family member. The variable predicting colonoscopy was receiving medical information regarding risk (odds ratio OR 8.40).

**Conclusions:**

We found that family cancer history characteristics (number of relatives with cancer, risk classification) are associated with adequate risk perception. Risk perception does not predict colonoscopy in this sample. The only variable that predicted colonoscopy was receiving medical information recommending screening.

## Background

According to the Brazilian National Cancer Institute (INCA) [1], colorectal cancer (CRC) is the third most common cancer in Brazil, with an estimated 28,000 new cases expected to be diagnosed in 2010 [1]. The main risk factors for CRC development are family history, age, and dietary habits [2]. In a meta-analysis, Butterworth et al. [3] identified 59 studies published from 1958 to 2004 on CRC risk with a grouped relative risk for CRC in individuals with family history of 2.14 (95% confidence interval (CI) 1.98-2.32). Hereditary conditions can be associated with an increase in risk of up to 100% [2].

Mortality associated with CRC can be reduced by early detection. A systematic review found that the use of the fecal occult blood test (FOBT) reduces the risk of CRC death by 16% [4]. A 15-year clinical study found that regular colonoscopies in high-risk individuals have reduced the incidence and mortality of CRC [5].

In 2003, INCA published recommendations for CRC screening with FOBT for individuals with a general population risk (50 years or older with no other additional risk factors), and colonoscopy for positive FOBT tests. This recommendation also indicates that individuals with a family history of CRC should be evaluated by a health professional to recommend testing for CRC early detection [6].

Although CRC is ideally suited for early detection measures, the willingness of individuals to adhere to screening recommendations is low. According to the 2000 National Health Interview Survey, only 45% of men and 41% of women aged 50 years or more reported completion of FOBT in the previous year or colorectal endoscopy in the last 10 years [7].

Because the participation is considered low, it is necessary to understand why individuals do not adhere to screening recommendations. Risk perception is among the factors cited in the literature as predictors of having a colonoscopy [8-10].

Collins et al. [8] evaluated 114 individuals tested for a predisposition to Lynch Syndrome and found that the only variable associated with colonoscopy before the test was the perception of risk (odds ratio (OR) 1.03; 95%CI 1.00-1.05). Risk perception, or susceptibility, refers to the subjective risk of an individual to develop a certain condition or disease [11-13]. Depending on the evaluation of the threat, it is considered a motivating factor for the adoption of preventive or protective behaviors [14,15]. It is thought that bringing risk perception and actual risk values closer enables the individual to adopt health behaviors appropriate to the level of risk [14,16]; however, this idea has yielded conflicting results in the literature [16].

In addition to studying the association between risk perception and health behavior, it is important to evaluate which aspects are associated with overestimation or underestimation of risk. Several characteristics have been associated with changes in risk perception, including gender [17,18], age [18], family history [18-20], and educational level [19].

It should be noted that the majority of studies refer to North American and European samples. Because of the scarcity of studies in Latin-American populations, this study aims (1) to verify which demographic characteristics and cancer family history are predictors of risk perception, and (2) to verify if risk perception is a predictor of colonoscopy adherence.

## Methods

This descriptive and cross-sectional study was performed at a teaching hospital in Sao Paulo, Brazil and was approved by the Institutional Review Board. The inclusion criteria were individuals older than 18 years of age without a personal history of cancer who were first-degree relatives of a patient with CRC who fulfilled the Bethesda criteria [21] or whose family met the Amsterdam criteria [22,23].

To identify the research participants, patients with CRC were selected through the institutional hereditary CRC registry that was established in 1992. Patients received a letter requesting authorization for the researcher to enter into contact with their first-degree relatives. Each consenting participant signed two copies of an informed consent form and provided a list of their relatives.

A telephone call was made to the potential research volunteers to verify participation in the research. After the first contact, they received an invitation letter and two copies of an informed consent form, and a telephone interview was then scheduled. The telephone interview followed a script that included a questionnaire with demographic characteristics and information pertaining to colonoscopy history. Risk perception measures were based on Clarke et al. [24], as follows:

•	numeric scale of population risk perception: Individuals must choose between 0% (meaning no risk of the individual to develop CRC) and 100% (absolute certainty that the individual will develop CRC) about the chance of an individual from the general population to develop CRC;

•	numeric scale of personal risk perception: Individuals must choose between 0% (meaning no risk of the individual's having CRC) and 100% (absolute certainty that the individual will have CRC) about the individual's own risk of developing CRC;

•	verbal scale of personal risk perception: Individuals must indicate their own risk of developing CRC, using a verbal scale (low risk, medium risk, high risk);

•	comparative verbal scale of personal risk perception: Individuals must indicate their own risk of having CRC compared with the general population risk, using a verbal scale (lower risk, same risk, higher risk).

The comparative verbal scale of personal risk perception was used to classify risk perception as adequate or inadequate. For individuals that estimated their risk perception as higher or very higher in the comparative scale they were classified as adequate. Individuals that estimated their risk perception as very low, low or average were considered with inadequate risk perception.

Colonoscopy compliance was measured considering risk classification. For individuals that belong into families that fulfilled the Amsterdam Criteria, colonoscopy compliance was considered adequate if they reported a colonoscopy in the past two years. For individuals whose family member fulfilled the Bethesda Criteria, colonoscopy compliance was considered adequate if they reported a colonoscopy in the past five years.

The t-test or ANOVA was used to identify the association between demographic characteristics and family history and risk perception variables [25]; the chi-square test was used for analyzing independent variables and categorical, dependent variables (risk perception or colonoscopy).

After selection of the independent variables with a statistical significance cut-off of 0.20, a multiple regression was done. A multiple linear regression was used for the continuous dependent variables and multiple logistic regression for the categorical, dependent variables. Tests with p values less than 0.05 were considered statistically significant [25]. A multiple logistic regression was also used to identify factors that predicted colonoscopy in this sample. SSPS version 15 was used for statistical analysis.

## Results

### Sample characteristics

A total of 152 patients were identified. Of these, 62 returned the informed consent and provided a list of names and contact information of family members (there were 70 non-respondents, 9 refusals, and 11 cases for which the address was not valid). The 62 patients indicated a total of 180 relatives. Of this total, 39 were not located (a letter was sent and there were three attempts made to reach them by phone), 16 refused, and 8 were excluded because they were identified as second-degree relatives after the pedigree analysis. Thus, the sample consisted of 117 participants.

Table 1 shows the sample characteristics. The participant mean age was 45.2 years (range 18-70). Most respondents were less than 50 years old (62.4%); most were female (74.4%); 41.9% had a college education; and 88.5% were Catholic. Most came from families who met the Amsterdam criteria (54.7%), and 79.5% reported that at least one family member had died from cancer. When asked whether they had received information about CRC risk, 89.7% answered yes, and 82.1% reported knowledge regarding colonoscopy. From the total, 44.4% colonoscopy compliance was considered adequate.

**Table 1:** Sample characteristics

Sample characteristics	N	%
*Age*		
Up to 50 years	73	62.4
50 years or more	44	37.6
*Gender*		
Female	97	74.4
Male	30	25.6
*Education level*		
Elementary	22	18.8
High-school	46	39.3
College	49	41.9
Family income(monthly)*		
Up to US$1,098	31	26.5
From US$1,098 to US$2,746	27	23.1
More than US$2,747	33	28.2
*Religion*		
Catholic/other	100	85.5
Evangelic/Protestant	9	7.7
*Risk classification*		
Bethesda criteria	53	45.3
Amsterdam criteria	64	54.7
*Death of a family member from colorectal cancer*		
No	24	20.5
Yes	93	79.5
*Received information regarding colorectal risk*		
No	12	10.3
Yes	105	89.7
*Colonoscopy compliance*		
No or Inadequate	65	55.6
Yes	52	44.4

Total	117	100.0

* 26 individuals did not respond.

#### Risk perception

The mean of population risk perception, according to numeric scale, was 47.6% with a median of 50%. The average perception of individual risk was 55.4% with a median of 55%.

Twenty-three (19.7%) individuals did not address risk perception with numerical scales. Factors that predicted response to risk perception evaluated by numerical scales were individuals 50 years or less (OR 3.30; 95%CI 1.26-8.69) and those with a college degree (OR 3.15; 95%CI 1.06-9.42).

Of the 115 individuals who responded to the question about risk perception with a verbal scale, 58.3% reported a high risk of developing CRC and 41.7% reported a low or medium risk. According to the comparative verbal scale, 69.0% reported higher risk, and 31% reported lower risk or the same (one respondent did not answer this question).

Table 2 presents the multiple linear regression results for the population risk perception according to the numerical scale and Table 3 for personal risk perception according to the numerical scale. The variables kept in the model were responsible for 18.8% of the variance in perception of population risk. The standardized regression coefficients indicate that female gender, elementary school education level, and family income lower than US$1,098/month were associated with an overestimation of population risk according to the numerical scale.

**Table 2:** Multiple linear regression model for population risk perception according to the numeric scale

Adjusted R2	Statistical significance of the model	Model variables	Standardized Coefficients (β)	p
0.188	<0.001	Education: elementary	0.249	0.017
		Gender: female	0.216	0.025
		Income: less than US$1,098	0.206	0.048

**Table 3:** Multiple linear regression model for personal risk perception according to the numeric scale

Adjusted R2	Statistical significance of the model	Model variables	Standardized coefficients (β)	p
0.285	<0.001	Amsterdam criteria: yes	0.332	0.001
		Religion: Catholic	0.261	0.007
		Gender: female	0.179	0.063
		Age: up to 50 years	0.149	0.124
		Received information: yes	0.142	0.145

Regarding personal risk perception according to the numerical scale, variables kept in the model were responsible for 28.5% of the variance. Variables associated with greater perception of personal risk were family history (fulfillment of the Amsterdam criteria) and religion (Catholic/other).

Table 4 presents the results of multiple logistic regression for individual risk according to the verbal scale. The verbal scale model accounted for 32.4% of the variance. Catholic/other religion, age (≤50 years), female gender, and family income (≥US$2,747/month) were associated with a higher risk perception according to the verbal scale.

**Table 4:** Multiple logistic regression for personal risk perception according to the verbal scale

Variable	OR*	95%CI	p
Religion			
Protestant	1		
Catholic/other	5.3	2.1-21.3	0.010
Age			
>50 years	1		
≤50 years	3.8	1.4-10.2	0.006
Gender			
Male	1		
Female	3.4	1.2-9.9	0.023
Family income			
< US$2,746	1		
≥ US$2,747	3.3	1.1-9.8	0.032
Relatives with cancer who died			
0	1		
1 or more	3.0	0.9-9.4	0.058

* OR: Ratio of chances refers to high individual risk according to the verbal scale.

Table 5 presents the results of multiple logistic regression for individual risk according to the comparative verbal scale. The comparative verbal scale model accounted for 28.1% of the variance. Death of a family member and female gender were associated with higher risk perception according to the comparative verbal scale.

**Table 5:** Multiple logistic regression for individual risk perception with the verbal and comparative verbal scales

Variable	OR*	95%CI	p
Relatives with cancer who died			
0	1		
1 or more	5.7	1.7-19.0	0.005
Gender			
Male	1		
Female	3.3	1.2-9.1	0.017
Family income			
< US$2,746	1		
≥ US$2,747	3.4	0.8-13.6	0.085
Education level			
Elementary/high-school	1		
College	1.9	0.6-5.9	0.255
Amsterdam criteria			
No	1		
Yes	1.6	0.6-4.3	0.326

* OR: Ratio of chances of individuals refers to high individual risk according to the verbal scale and higher individual risk according to the comparative verbal scale.

The risk perception was considered inadequate in 32 (27.4%) of the participants and adequate in 85 (72.6%) of the participants. The only variable that predicted adequate risk perception was the number of family members with cancer; having three or more family members with CRC resulted in more accurate risk perception (OR 4.72 p 0.023).

#### Risk perception and colonoscopy

Risk perception was associated with colonoscopy only when measured with the comparative scale. Of the 36 individuals who reported having a lower or the same risk as individuals from the general population, 16 (45%) had undergone a colonoscopy, while 54 (67.9%) of the 80 individuals who reported higher risk had undergone the procedure (p = 0.015).

Based on the multivariate analysis, risk perception did not predict colonoscopy. The only variable that predicted colonoscopy was receiving medical information recommending screening (OR 8.40; 95%IC 1.55-48.71).

## Discussion

In the search for the motives that lead some individuals to have an exam and others to choose not to, an increasing number of studies since the 1950s have evaluated health behaviors related to cancer prevention. This study is one of the first to evaluate CRC-related health behaviors in Brazil.

The assessment of health behaviors is important in different cultures, as are differences in risk perception. Kim et al. [26] evaluated 1160 women with perceived risk of cancer of the breast, cervix, and colon and noted that Latinas had a perception of higher risk compared to Whites, African-Americans, and Asians.

Although there is no screening program for CRC in Brazil, some institutions have established programs for high-risk individuals. Understanding the beliefs of high-risk individuals may contribute to establishing educational and psychosocial interventions that take into account not only the technical and scientific knowledge of health professionals but also the beliefs and attitudes of lay people.

The low response frequency of solicited patients to indicate their relatives (40.8%) should be noted. One of the possible explanations for this outcome is that patients did not want their relatives to be contacted in order to preserve the family. Koehly et al. [27], evaluating the communication pattern of five Lynch Syndrome families, verified that communication among members of the family depends on the psychosocial characteristics of their relationships [27].

Regarding the number of individuals who responded to questions on risk perception, a lesser number of individuals responded to questions about risk perception using a numeric scale as compared to questions using a verbal scale. This fact may highlight the difficulties individuals experience in estimating using a numeric scale. Although this study did not assess numeracy, these results point to the need to do so; according to Keller and Siegrist [28], different formats in the communication of risk perception produce different effects on risk perception, and numeracy mediates these differences. Kelly et al. [29] evaluated the numeric comprehension of women and their relation to risk perception and verified that younger women with more education presented greater numeric comprehension.

Individuals in this study overestimated the CRC population risk. An overestimation may reflect a lack of numeric ability, an influence of experiences with cancer, or a coping mechanism.

According to Price et al. [30], to estimate individual risk, the individual uses personal characteristics and behavior. To estimate population risk, the individual identifies the frequency of the event in the population, and the belief of individual risk does not depend on the perception of frequency of the event in the population. Individuals who are considered high risk, based on their judgment of personal characteristics, may judge that an event is relatively frequent [30].

The median of population risk and numeric scale individual risk in this study was 50%. Klein and Stefanek [31] highlighted the difficulties individuals experience in establishing strategies to cope with numeric information and pointed out that "50%" may not be used in numeric form but as an expression of doubt about the occurrence of an event, especially to estimate their own risk in an uncertain situation [31]. Risk perception is considered an essentially cognitive evaluation, and it is believed that the perception of risk also includes emotional reactions, especially with a verbal scale [32,33]. According to Bottorf et al. [34], the conversion of numeric information to a verbal scale (low, medium, or high risk) is a mechanism to simplify complex information in a way that facilitates the decision process.

Women reported a perception of greater population risk than men. Health perception in women is influenced by context, not only personal context but also that of others around them. Because women traditionally assume caring roles, contact with the experiences of relatives is greater and as such leads to more reaction to events than men may have [35,36].

Lower income was associated with overestimation of the risk population. This may reflect the socioeconomic status, which may indicate that individuals with less lower income have more difficulty in interpreting numerical information. This assumption may also explain why people with higher incomes reported greater perception of risk in accordance with the verbal scale.

Despite the fact that mutation analysis was not performed in this sample, since all participants had at least one family member with CRC diagnosed before 45 years old, we considered those individuals with a higher risk of CRC when compared with general population. However, 27.4% of this sample indicated their risk as lower or the same than general population. The only variable that predicted adequate risk perception was the number of family members with cancer which may suggest that family experiences with the disease can modulate risk perception.

Risk perception did not predict colonoscopy. However, medical advice was a factor in the performance of colonoscopy, as other studies have found [37-39]. Therefore, health care professionals have an important role informing individuals regarding the need to be submitted to a colonoscopy.

This study has some limitations. It is a cross-sectional study and therefore did not assess changes in risk perception over time. Moreover, the selection of participants occurred from the indication of the proband. This procedure was adopted because of ethical aspects associated with the fact that we had no direct initial contact with family members, only with patients. However, this study is one of the first done in Brazil to evaluate health behaviors in individuals at high risk for CRC.

## Conclusions

We found that family cancer history characteristics (number of relatives with cancer, objective risk classification) are associated with adequate risk perception. Risk perception does not predict colonoscopy in this sample. The only variable that predicted colonoscopy was receiving medical information recommending screening.

## List of Abbreviations

CI: Confidence interval; CRC: Colorectal cancer; FOBT: fecal occult blood test; INCA: Brazilian National Cancer Institute; OR: Odds Ratio

## Competing interests

The authors declare that they have no competing interests.

## Authors' contributions

EMMS: conceived of the study, interviewed participants and performed the statistical analysis. MTCL: participated in the study design and helped to draft the manuscript. BMR: participated in the study design and helped to draft the manuscript. All authors read and approved the final manuscript.
